# Seasonal dynamics of *Haemaphysalis* tick species as SFTSV vectors in South Korea

**DOI:** 10.1128/spectrum.00489-24

**Published:** 2024-09-30

**Authors:** Hyunwoo Jang, Mark Anthony B. Casel, Seung-gyu Jang, Jeong Ho Choi, Juryeon Gil, Rare Rollon, So youn Cheun, Young-Il Kim, Min Suk Song, Young Ki Choi

**Affiliations:** 1 College of Medicine and Medical Research Institute, Chungbuk National University, Cheongju, South Korea; 2 Center for Study of Emerging and Re-emerging Viruses, Korea Virus Research Institute, Institute for Basic Science (IBS), Daejeon, South Korea; Uniwersytet Medyczny w Bialymstoku, Bialystok, Poland

**Keywords:** severe fever with thrombocytopenia syndrome virus (SFTSV), *Haemaphysalis flava*, *Haemaphysalis longicornis*, prevalence, diagnosis

## Abstract

Ticks pose a significant public health threat due to their ability to transmit various pathogens, including emerging tick-borne diseases. This study conducted a comprehensive surveillance of *Haemaphysalis* tick species and their severe fever with thrombocytopenia syndrome virus (SFTSV) infection rates in South Korea throughout the year 2023, from January to December. To ensure accurate and rapid identification of the prevalent *Haemaphysalis* tick species in South Korea, we designed PCR primer sets targeting the ITS1 gene, specifically distinguishing *Haemaphysalis longicornis* from *Haemaphysalis flava*. Among the 10,343 ticks collected from wild animals, *H. longicornis* constituted the majority, accounting for 65.5% (6,784/10,343 ticks), followed by *H. flava* at 33.8% (3,491/10,343 ticks), and *Ixodes nipponensis* at 0.7% (68/10,343 ticks). These identified ticks were then categorized into 811 pools, with 63 pools testing positive for SFTSV. Remarkably, the prevalence of SFTSV-positive *H. longicornis* ticks peaked during the summer months, aligning with heightened human outdoor activities and, consequently, an increased risk of human exposure. Conversely, it is noteworthy that *H. flava* exhibited a higher prevalence during the winter season, reaching its peak in January, with an SFTSV minimum infection rate similar to that of *H. longicornis*. These findings underscore the year-round presence of *Haemaphysalis* ticks as potential vectors for SFTSV, extending the temporal window for potential human exposure. Consequently, these results emphasize the necessity for active and continuous field surveillance to comprehensively understand and mitigate the public health risks associated with these tick-borne pathogens.

**IMPORTANCE:**

To date, the majority of tick surveillance studies have primarily focused on warmer seasons, which are considered optimal periods for ticks to actively seek hosts and transmit pathogens through blood-feeding activities. Consequently, tick species active during winter have often been overlooked, leading to an underestimation of their significance in transmitting severe fever with thrombocytopenia syndrome virus (SFTSV). In this study, we aimed to examine year-round tick prevalence with SFTSV and illuminate the role of the winter-dominant species, *Haemaphysalis flav*a, in South Korea. Through rigorous identification facilitated by a primer set designed specifically for this purpose, we emphasize that *H. flava*, a competent vector species, harbors SFTSV in the winter season, thereby acting as an overwintering reservoir for the virus. This phenomenon may contribute to a higher infection rate among ticks in the following year.

## INTRODUCTION

Emerging zoonotic vector-borne diseases pose a relentless threat to global public health, leading to deadly outbreaks worldwide. Among these, diseases associated with tick-mediated transmission are of significant concern. Ticks, as hematophagous arthropods, parasitize a wide range of hosts and are vectors for diverse pathogens critical to human and animal health, including various bacteria and viruses. They often harbor multiple pathogens concurrently (1). Numerous attributes of ticks have been underscored in relation to their vector competence, encompassing their feeding behaviors, extended longevity, resilience to extreme environmental conditions, and notably their ability for transovarial and transstadial pathogen transmission (2).

Severe fever with thrombocytopenia syndrome virus (SFTSV) or *Bandavirus dabieense,* as designated by the International Committee on Taxonomy of Viruses (3), is a notable emerging tick-borne virus responsible for severe fever with thrombocytopenia syndrome (SFTS). It was first reported in China in 2009 (4) and subsequently in Korea and Japan in 2013 (5, 6). SFTSV has recently been identified in Vietnam, Myanmar, Pakistan, and Thailand (7–10). SFTS typically presents with severe clinical symptoms and has a case fatality rate of 12%–30% (4, 11). In Korea, the continuous rise in annual SFTSV cases since its first detection in 2013 underscores its significance (5). As a zoonotic disease, ticks play a pivotal role in SFTSV transmission among vertebrate hosts, including humans (4). Several hard tick species, such as *Haemaphysalis longicornis, Rhipicephalus microplus, Amblyomma testudinarium,* and *Ixodes nipponensis*, have been identified as carriers of SFTSV RNA (12–14). Notably, *H. longicornis* is the most prevalent tick species in South Korea, followed by *H. flava* (15, 16). Despite tick’s critical role in SFTSV dissemination, limited research has focused on their function as natural reservoirs and in maintaining the virus in the environment. The detection of SFTSV in field-collected and livestock-associated ticks poses a significant risk to human and animal populations in affected areas.

Although previous studies have explored tick prevalence and viral infections in an attempt to capture the natural tick activity period, however, many have focused on specific seasons, typically spring to fall. Herein, we collected ticks directly from wild animals year-round, from January to December 2023, and investigated the occurrence of SFTSV and the true seasonal prevalence of *Haemaphysalis* ticks harboring the virus in South Korea. To accurately identify the two predominant domestic tick species, *H. longicornis* and *H. flava*, we developed specific primers targeting the internal transcribed spacer 1 (ITS1) gene for molecular species identification to resolve morphological ambiguities between these species. Unlike conventional molecular analysis, which relies on the cytochrome c oxidase subunit 1 (COI) gene and often requires sequence analysis due to indistinguishable amplicon sizes between tick species (17), our primers were meticulously designed to amplify ITS1 gene segments of varying sizes unique to each of the two tick species under investigation. Our findings revealed distinctive seasonality in the prevalence of SFTSV-positive *Haemaphysalis* ticks, and *H. longicornis* exhibited a peak in prevalence during the summer season, coinciding with the surge in human outdoor activities, increasing the risk of human exposure to SFTSV. Conversely, *H. flava* predominated during the winter season among wild animals; however, both species displayed a comparable SFTSV minimum infection rate (MIR). These results provide evidence of the year-round presence of *Haemaphysalis* ticks as potential vectors for SFTSV, emphasizing the importance of active and continuous field surveillance to gain deeper insights and mitigate the public health risks associated with these tick-borne pathogens.

## MATERIALS AND METHODS

### Tick collection and identification

In this study, ticks were systematically collected from water deer (*Hydropotes inermis*) and wild boars (*Sus scrofa*), both of which are designated as pests under South Korean law. The hunting activities were carried out under the supervision of the Wildlife Management Association, in accordance with all relevant legal regulations and ethical considerations. All wild animals were euthanized humanely in compliance with established protocols. The carcass was positioned on a white cloth to facilitate the collection of ticks that emerged following the host’s demise, with particular attention paid to the ear, neck, and axillary regions through meticulous observation. Following collection, ticks were segregated by species and date per area. Morphological identification was performed using a stereomicroscope (Olympus, Tokyo, Japan), guided by established taxonomic keys (18). Larval ticks were excluded from identification and viral detection analyses. For molecular identification, the hind leg of each tick was excised, lysed, and subjected to DNA extraction using the DNeasy kit (Qiagen, Hilden, Germany) as per the manufacturer’s instructions. The ITS1 gene’s partial nucleotide sequences were amplified with specific primers (ITS1_FL_F, ITS1_F_R, ITS1_L_R) (Table 1). PCR conditions were as follows: initial denaturation at 94°C for 5 min, followed by 35 cycles of 94°C for 30 s, 60°C for 45 s, 72°C for 1 min, and a final extension at 72°C for 7 min. To clarify the cross-reactivity of this primer, following the same protocol aforementioned, extracted DNA from the hind leg of *I. nipponensis* and blood from water deer and wild boar were used as control. In addition, extracted DNA was validated by amplifying each of the COI following the thermocycling program: 94°C for 5 min, followed by 35 cycles of 94°C for 30 s, 58°C for 30 s, 72°C for 1 min, and a final extension at 72°C for 7 min for both water deer and wild boar and 94°C for 5 min, 35 cycles at 94°C for 1 min, 53°C for 1 min, and 72°C for 1 min, followed by a final extension at 72°C for 8 min for the *I.nipponensis*.

**TABLE 1:** Polymerase chain reaction primers used in this study

Target gene	Primer	Primer sequence (5’–3’)	Amplicon size(bp)	Reference
*H. flava* and *H. longicornis* ITS1^*a*^	ITS1_FL_F	GGT CGG CCC TCG ATT CCA	521 bp	In this study
	ITS1_F_R	ACG CAG ACC CTG GCT TTC CTA		
	ITS1_L_R	TCT CCC GTC GCT ATC GCC	1021 bp	
Hard tick COI^*b*^	LCO1490	GGT CAA CAA ATC ATA AAG ATA TTG G	707 bp	(16)
	HCO2198	TAA ACT TCA GGG TGA CCA AAA AAT CA		
*Hydropotes inermis* COI^*b*^	*H.inermis*_F	GTG CTC CAG ATA TAG CAT TTC CTC G	434 bp	In this study
	*H.inermis*_R	TTG ATA TAG GAT AGG GTC TCC GCC		
*Sus scrofa* COI^*b*^	*S.scrofa_F*	GCT CAC CAC ATA TTC ACC GT	462 bp	In this study
	*S.scrofa_R*	AAT CGG AGT ATC GTC GAG GT		
SFSTV M Segment	M_805F	ACT GGG CCG TGT TCT GAA TCA GA	604 bp	In this study
	M_1409R	TAT CCA AGG AGG ATG ACA ATA AT		
	M_811F	GAA GAG ACT TGC AAG ACC AGT GGT	429 bp	
	M_1240R	CCA CCA AAG CTC CTT TGA CGT GTA		

^*a*^ ITS1: Internal transcribed spacer 1.

^*b*^ COX: Cytochrome c oxidase subunit 1.

### RNA extraction and SFTSV detection

The collected ticks were pooled into microcentrifuge tubes based on host, collection area, date, developmental stages, and species (10 adult ticks/tube, 20 nymphs/tube). The tubes were supplemented with 650 µL of 60% antibiotics [a mixture of polymyxin B sulfate salt, gentamycin, nystatin, ofloxacin, and sulfamethoxazole in Eagle's minimum essential medium (EMEM, Lonza, Basel, Switzerland)] and homogenized using sterile 3 mm Tungsten Carbide beads (Qiagen, Hilden, Germany) at 30 Hz for 5 min. Post-centrifugation at 13,000 × *g* for 5 min, 250 µL of supernatant was used for RNA extraction using TRIzol reagent (Invitrogen, Carlsbad, CA, USA) and further purified with the RNeasy kit (Qiagen, Hilden, Germany). For SFTSV detection, reverse transcription was performed to synthesize cDNA using Superior Script III Reverse Transcriptase (Enzynomics, Daejeon, South Korea). The cDNA served as a template for primary PCR amplification of the SFTSV M gene followed by nested PCR with the primer designed in this study (M_805F, M_1409R and M_811F, M_1240R for primary and nested PCR) (Table 1). Both primary and nested PCR shared the following conditions: 94°C for 5 min, 35 cycles of 94°C for 30 s, 58°C for 30 s, 72°C for 30 s, and a final extension at 72°C for 7 min. Amplified products were visualized on 1% agarose gels. Positive amplicons were then purified using Expin Gel SV (GeneAll, Seoul, Korea) according to the manufacturer’s instructions and directly sequenced using a 3730XL DNA analyzer (Applied Biosystems, Foster City, USA) at Bionics Co., Ltd (Seoul, Korea). Sequences were analyzed and compiled with DNA Star 5.0 (DNASTAR, Madison, WI, USA); closely related viruses were identified using the Basic Local Alignment Search Tool (https://blast.ncbi.nlm.nih.gov/Blast.cgi).

### Phylogenetic analysis

Partial M segments detected in this study were aligned with the 38 SFTSV M segments including A–F genotypes of SFTSV, which are available in GenBank (National Center for Biotechnology Information, NCBI, Bethesda, MD, USA). Multiple sequence alignments were performed using the ClustalW algorithm in MEGA 6.0 software (MEGA6: molecular evolutionary genetics analysis version 6.0) as previously described (19). Phylogenetic trees were constructed using the maximum likelihood (ML) method based on Kimura’s 2-parameter model, with tree reliability assessed by a bootstrap test with 1,000 replications.

## RESULTS

### Molecular discrimination of *Haemaphysalis* tick species: *H. longicornis* and *H. flava*

**Fig 1:**
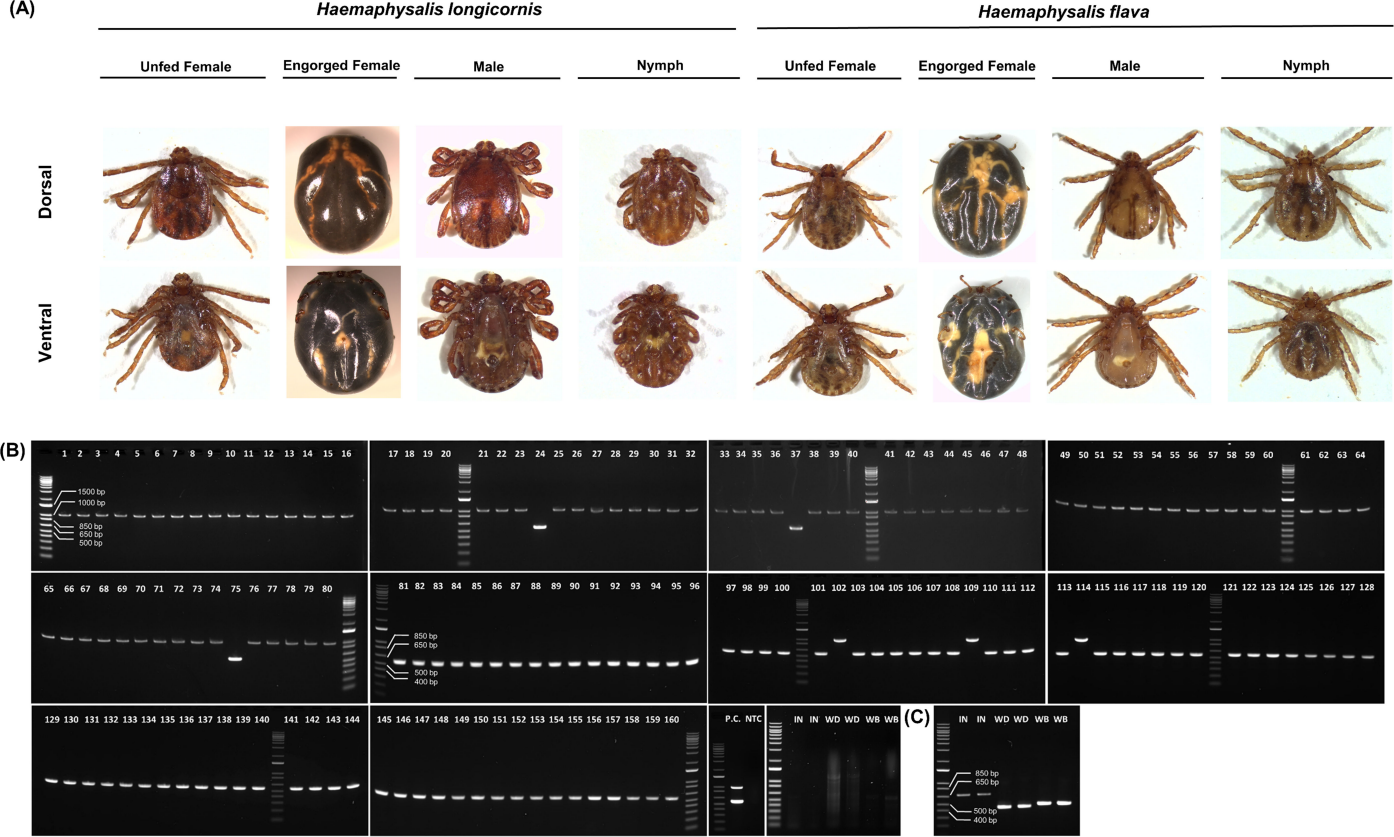
Photograph of *H. longicornis* and *H. flava* collected from wild animals and agarose gel electrophoresis with PCR amplification products of ITS1 and COI genes. (**A**) Dorsal (up) and ventral view (down) of *H. longicorn*is and *H. flava* in different developmental stages or physiological statuses. (**B**) Molecular species identification targeting ITS1 gene of *H. longicornis* (1,021 bp) and *H. flava* (521 bp). Numbers indicate morphologically identified species as follows: 1–20: unfed female *H. longicornis* adult, 21–40: engorged female *H. longicornis* adult, 41–60: male *H. longicornis* adult, 61–80: *H. longicornis* nymph, 81–100: unfed female *H. flava* adult, 101–120: engorged female *H. flava* adult, 121–140: male *H. longicornis* adult 141–160: *H. longicornis* nymph, P.C. : positive control mixed with *H. longicornis* and *H. flava* DNA extracts, and NTC : negative template control. To examine the specificity of the primer designed in this study, the following DNA extracts were added as a control. IN: *I. nipponensis*, WD: water deer (*Hydropotes inermis*), and WB: wild boar (*Sus scrofa*). (**C**) Amplification targeting COI of *I. nipponensis* (IN), water deer (WD), and wild boar (WB), respectively.

Given their prevalence and morphological similarities, the precise identification of *Haemaphysalis* species, especially *H. longicornis* and *H. flava*, is critical for comprehensively understanding the eco-epidemiology of the virus and the dynamics of vector and reservoir species. To differentiate between these two prevalent domestic tick species, we developed species-specific primers targeting the ITS1 gene to resolve the morphological similarities between *H. longicornis* and *H. flava*. The primer design was based on ITS1 gene alignments, aiming to amplify species-specific conserved regions with distinctive PCR product sizes: 1,021 bp for *H. longicornis* and 521 bp for *H. flava*. PCR amplicons underwent sequence analysis using the NCBI Blast tool, which confirmed a high degree of sequence identity (98%–100%) specific to each species (Table S1). The assay’s specificity was further validated by testing non-target DNA samples from *I. nipponensis*, water deer, and wild boar, which were successfully amplified using the COI targeting primer of each species. This confirms the reliable discrimination between the two tick species through variations in the length of PCR amplification of the ITS1 gene (Fig. 1).

To assess the accuracy of distinguishing between the two *Haemaphysalis* tick species using the morphological and molecular identification methods, we examined a total of 160 ticks, including unfed females (*n* = 20), engorged females (*n* = 20), male adults (*n* = 20), and nymphs (*n* = 20) from each species. These ticks were selected regardless of their collection date and initially identified using a stereomicroscope. Subsequently, the ticks that were morphologically identified underwent DNA extraction, followed by PCR amplification using the primers specified in this study (ITS1_FL_F, ITS1_F_R, and ITS1_L_R) (Table 1). Although the molecular diagnostic analyses consistently aligned with the morphological identification for unfed adult ticks of the two *Haemaphysalis* species, a decrease in accuracy was observed among engorged and nymph samples. This reduction in accuracy can primarily be attributed to the distension of the tick body after blood-feeding, which alters their original anatomical features. Additionally, partial damage to the anterior parts of nymphs may have occurred due to improper sampling collection (Fig. 1; Table 2).

**TABLE 2:** Evaluation of morphological identification precision compared with molecular identification based on the ITS1 gene

	*H. longicornis*				*H. flava*			
Developmental stage	Unfed female	Engorged female	Male	Nymph	Unfed female	Engorged female	Male	Nymph
Test no.	20/20	18/20	20/20	19/20	20/20	17/20	20/20	20/20
Accuracy rate	100%	90%	100%	95%	100%	85%	100%	100%

### Temporal distribution of biological vector

**Fig 2:**
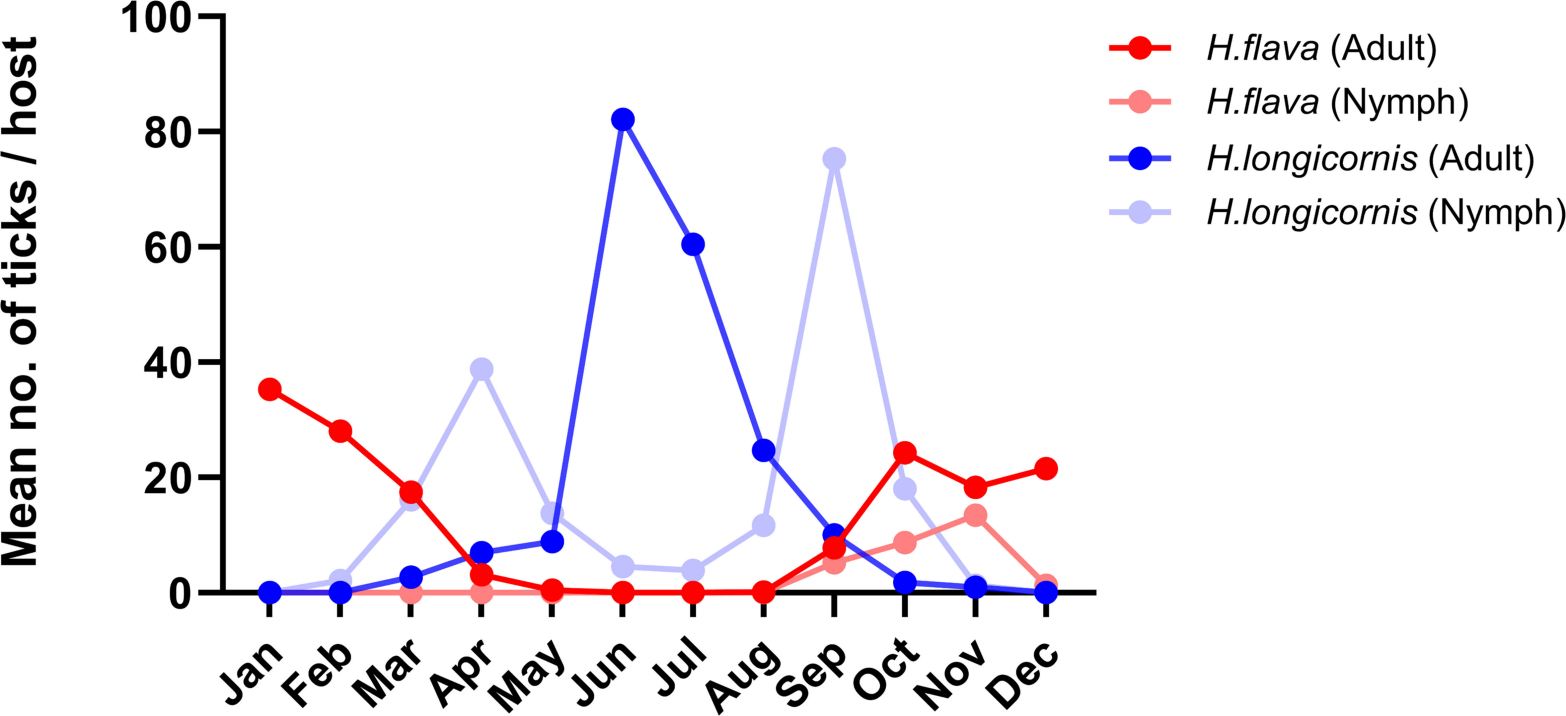
Year-round occurrences of *H. longicornis* and *H. flava* in South Korea from January to December 2023. The average number of collected ticks per host is presented by tick species and developmental stages. The red and pink colors represent the adult and nymphal stages of *H. flava*. Blue and light blue indicate *H. longicornis* adult and nymph, respectively.

During the entire study period, spanning from January to December 2023, a total of 10,343 ticks were meticulously collected from 197 water deer and 35 wild boars (Table S2). Each tick was initially identified by species based on the collection date, with subsequent molecular analyses confirming these identifications. The findings revealed that *H. longicornis* was the most predominant species, comprising 65.5% of the total collection (*n* = 6,784), followed by *H. flava* at 33.8% (*n* = 3,491) and *I. nipponensis* at 0.7% (*n* = 68) (Table 3). Notably, the temporal distribution of *Haemaphysalis* species exhibited significant variation. *H. longicornis* was most abundant from April to September, followed by a gradual decline from October to November, and was not detected from December to January. Conversely, the population of *H. flava* gradually increased starting in September, peaked from December to January, and then decreased in February (Fig. 2; Table 3; Table S3). These observations underscore the distinct seasonal patterns in the prevalence of *H. longicornis* and *H. flava*. Although *I. nipponensis* comprised only 0.7% of the total collection, adult specimens of this species were predominantly found from October to December, suggesting a seasonal prevalence pattern akin to that of *H. flava*. Notably, these findings collectively indicate the continuous presence of *Haemaphysalis* ticks as potential vectors for SFTSV throughout the year.

**TABLE 3:** Total ticks collected from wild animals in South Korea from January to December 2023

Species	Stage	Number of collected ticks and pools from South Korea												
		Jan	Feb	Mar	Apr	May	Jun	Jul	Aug	Sep	Oct	Nov	Dec	Total
*H. longicornis*	Adult	0 (0)^*a*^	0 (0)	49 (3)	118 (8)	248 (25)	1,232 (120)	1,512 (132)	469 (45)	110 (10)	35 (6)	25 (3)	0 (0)	3,798 (352)
	Nymph	0 (0)	40 (4)	290 (10)	660 (29)	387 (18)	68 (3)	96 (8)	222 (13)	828 (44)	361 (23)	34 (2)	0 (0)	2,986 (154)
*H. flava*	Adult	494 (47)	533 (38)	314 (18)	53 (4)	13 (1)	0 (0)	0 (0)	0 (0)	86 (9)	485 (49)	476 (49)	430 (47)	2,884 (262)
	Nymph	0 (0)	0 (0)	0 (0)	0 (0)	0 (0)	0 (0)	0 (0)	0 (0)	57 (3)	174 (9)	350 (18)	26 (2)	607 (32)
*I. nipponensis*	Adult	0 (0)	11 (2)	0 (0)	0 (0)	0 (0)	0 (0)	0 (0)	0 (0)	0 (0)	10 (3)	31 (4)	12 (1)	64 (10)
	Nymph	0 (0)	0 (0)	0 (0)	4 (1)	0 (0)	0 (0)	0 (0)	0 (0)	0 (0)	0 (0)	0 (0)	0 (0)	4 (1)
Total		494 (47)	584 (44)	653 (31)	835 (42)	648 (44)	1300 (123)	1608 (140)	691 (58)	1081 (66)	1065 (90)	916 (76)	468 (50)	10343 (811)

^*a*^ Numbers in parentheses indicate the number of pools.

### Minimum infection rate and seasonal distribution of SFTSV-positive ticks

**Fig 3:**
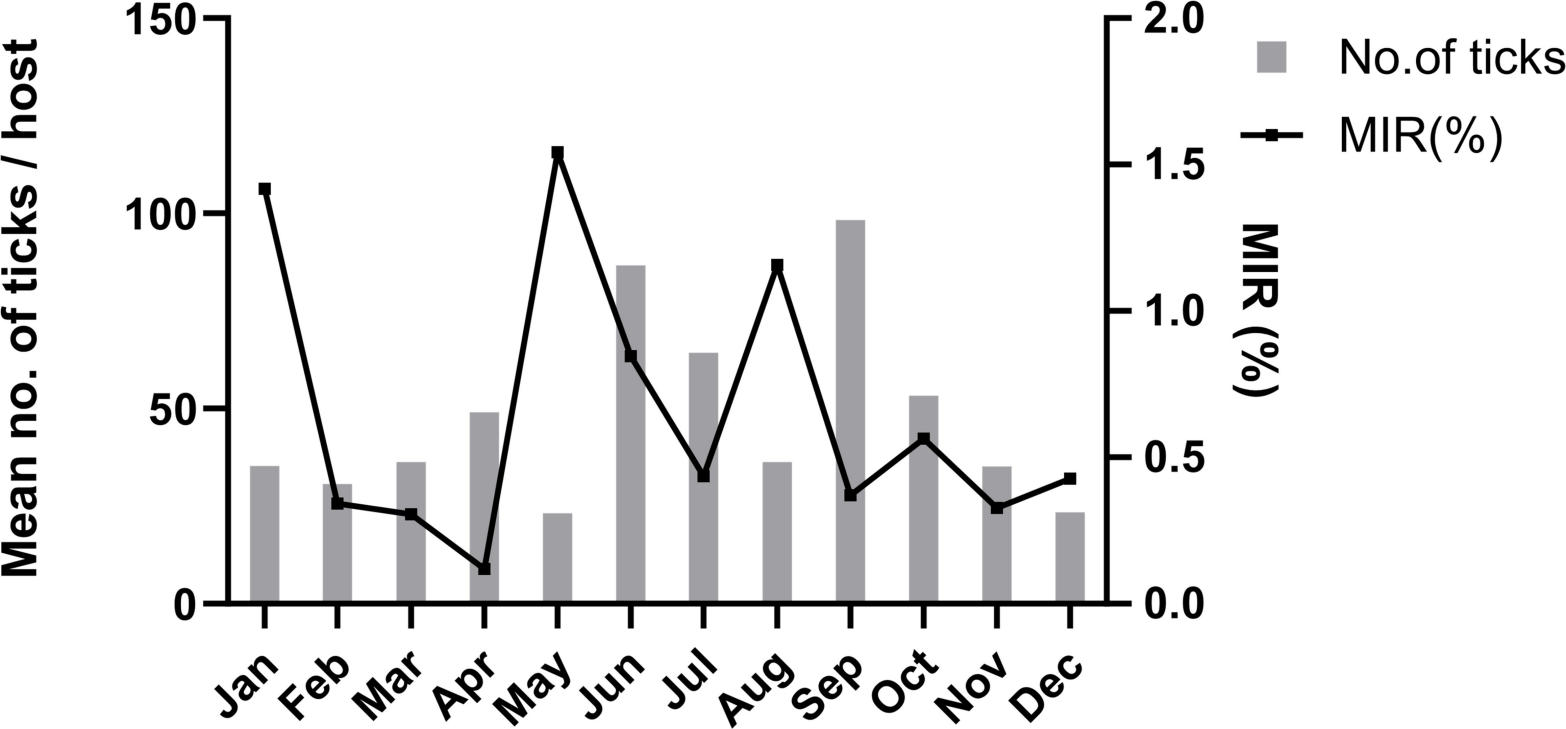
Year-round occurrences of *H. longicornis* and *H. flava* in South Korea from January to December 2023. The average number of collected ticks per host is presented by tick species and developmental stages. The red and pink colors represent the adult and nymphal stages of *H. flava*. Blue and light blue indicate *H. longicornis* adult and nymph, respectively.

A total of 10,343 ticks were categorized into 811 pools based on their developmental stages, collection dates, hosts, and geographic locations. Nested PCR analysis, employing the primer set designed in this study (Table 1), was utilized to calculate the overall MIR, defined as the ratio of the number of positive pools to the number of tested ticks, multiplied by 100, for SFTSV throughout the entire collection period in South Korea. The calculated MIR for SFTSV across South Korea was 0.6% (Table 4). Among the 63 SFTSV-positive tick pools, 41 were attributed to *H. longicornis* and 22 to *H. flava*. The cumulative MIR for *H. longicornis* was 0.6%, whereas for *H. flava,* it was 0.63%, with notably higher MIRs recorded for *H. flava* in January (1.41%) and for *H. longicornis* in May (1.57%) (Table 4). Notably, SFTSV positivity rates were as high as 1.55% in nymphs and 1.61% in adults of *H. longicornis*. In contrast, *H. flava* exhibited a MIR of 0.57% in nymphs and 1.41% in adults. The peak MIRs for SFTSV across all tick species collected in this study were observed in January (1.41%), May (1.54%), and August (1.15%) (Fig. 3).

**TABLE 4:** Minimum infection rate (%) of SFTSV in ticks collected from wild animals in South Korea from January to December 2023

Species	Stage	Monthly minimum infection rate of collected ticks from South Korea												
		Jan	Feb	Mar	Apr	May	Jun	Jul	Aug	Sep	Oct	Nov	Dec	Total
*H. longicornis*	Adult	0	0	0	0	1.61	0.89	0.46	1.49	0	0	0	0	0.76
	Nymph	0	0	0.34	0.15	1.55	0	0	0.45	0.36	0	0	0	0.40
	Total	0	0	0.29	0.12	1.57	0.84	0.43	1.15	0.31	0	0	0	0.60
*H. flava*	Adult	1.41	0.37	0.31	0	0	0	0	0	0.69	0.91	0.36	0.43	0.69
	Nymph	0	0	0	0	0	0	0	0	0	0.57	0.28	0	0.32
	Total	1.41	0.37	0.31	0	0	0	0	0	0.69	0.91	0.36	0.43	0.63
*I. nipponensis*	Adult	0	0	0	0	0	0	0	0	0	0	0	0	0
	Nymph	0	0	0	0	0	0	0	0	0	0	0	0	0
	Total	0	0	0	0	0	0	0	0	0	0	0	0	0
Total		1.41	0.34	0.3	0.11	1.54	0.84	0.43	1.15	0.37	0.56	0.32	0.42	0.60

### Phylogenetic analysis

**Fig 4:**
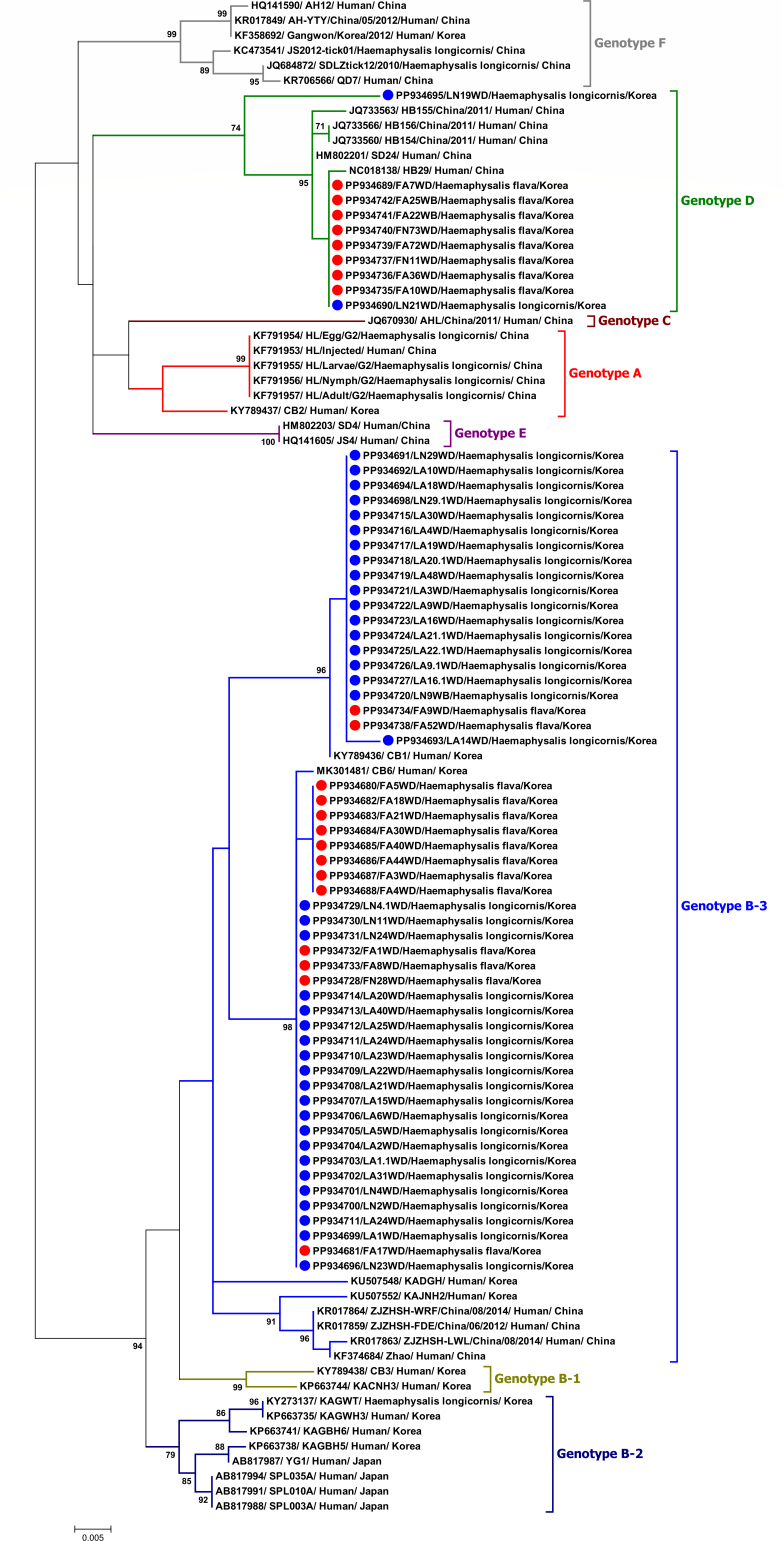
Phylogenetic analysis of SFTSV strains based on the M segment sequences. Phylogenetic analysis was performed using the maximum likelihood (ML) method with the Kimura 2-parameter model. Bootstrap support values based on 1,000 replicates are shown above the branch, and the phylogenetic branches were supported with greater than 70%. The color of the branch indicates the genotypes of the SFTSV strains. Partial sequences that were obtained in this study were marked with red and blue circles for *H. flava* and *H. longicornis,* respectively.

Phylogenetic analysis of 63 partial M gene sequences of SFTSV (GenBank Accession number: PP934680-PP934742) revealed distinct clustering patterns: 53 sequences (GenBank Accession number: PP934680, PP934681, PP934682, PP934683, PP934684, PP934685, PP934686, PP934687, PP934688, PP934691, PP934692, PP934693, PP934694, PP934696, PP934697, PP934698, PP934699, PP934700, PP934701, PP934702, PP934703, PP934704, PP934705, PP934706, PP934707, PP934708, PP934709, PP934710, PP934711, PP934712, PP934713, PP934714, PP934715, PP934716, PP934717, PP934718, PP934719, PP934720, PP934721, PP934722, PP934723, PP934724, PP934725, PP934726, PP934727, PP934728, PP934729, PP934730, PP934731, PP934732, PP934733, PP934734, and PP934738) predominantly formed a cluster within Genotype B-3, whereas the remaining 10 sequences (GenBank Accession number: PP934689, PP934690, PP934695, PP934735, PP934736, PP934737, PP934739, PP934740, PP934741, and PP934742) grouped with Genotype D (Fig. 4). Remarkably, although both genotypes were identified in both tick species, Genotype B-3 was primarily associated with *H. longicornis*, whereas Genotype D was predominantly detected in *H. flava*.

## DISCUSSION

Accurate identification of tick species is crucial for effective tick monitoring, population management, and control of tick-borne diseases. However, species identification, often relying on morphological characteristics, presents challenges, especially in the case of the *Haemaphysalis* genus, which is the second-largest within the Ixodidae family with over 160 species worldwide (20). Morphological differentiation, particularly among engorged and larval stages, can be challenging. Additionally, potential tick damage can complicate precise identification, making molecular identification indispensable. Previous studies have employed various methods primarily to distinguish *H. longicornis* from other *Haemaphysalis* species (21–23). In our study, we aimed to develop a reliable method that bypasses the need for detailed morphological analysis or extensive DNA sequencing. Leveraging the variability of the ITS1 gene locus (24, 25), we employed it for molecular analysis. The primer sets designed for the non-conserved ITS1 region between *H. longicornis* and *H. flava* successfully differentiated the two species with high specificity. These primers, tested against common Korean tick species (*I. nipponensis*, water deer, and wild boar DNA) showed no cross-reactivity. This primer set plays a crucial role in determining the distribution of tick species, assessing the prevalence of SFTSV and other tick-borne viruses, and conducting ecological studies on *H. longicornis* and *H. flava*. Additionally, this method allows for prompt and accurate molecular identification of ticks, including those in various conditions such as engorged, impaired, and larvae.

We assessed the temporal distribution of tick species and the MIR of SFTSV in South Korea, focusing on the key vectors *H. longicornis* and *H. flava*. The collection peak per host occurred in June and September, mirroring the species-specific trends. *H. longicornis* numbers peaked in these months, whereas *H. flava* emerged in September, increasing through winter. This indicates that *H. longicornis* is more active in warmer seasons (April to September), whereas *H. flava* is more prevalent in colder months (October to March). The surveillance study revealed peak SFTSV MIRs in January, May, and August, with distinct seasonal SFTSV prevalence of each tick species; *H. flava* showed the highest MIR in January and *H. longicornis* in May. Despite the different seasonal distributions, SFTSV positivity rates did not significantly vary between the species (0.6% in *H. longicornis* and 0.63% in *H. flava*). The higher prevalence of *H. flava* observed in this study, particularly during winter, highlights its potential role as an active SFTSV reservoir, counterbalancing the lack of winter data in previous Korean studies (15, 16, 26, 27).

Referencing Perez-Eid (28), we note that tick-borne encephalitis virus (TBEV) titers in non-diapause nymphal ticks exceed those in diapausing ticks, suggesting that diapause influences tick-virus interactions, potentially impacting SFTSV survival in dormant ticks. In addition, overwintering poses challenges to arthropod vectors, with *H. longicornis* experiencing field mortality rates from 9% to 40% (29), potentially leading to the loss of both vectors and viruses. However, our observations indicate that *H. flava* remains active in winter. Although tick mobility is limited in colder months, the virus’s ability to infect different tick species with unique seasonal distributions allows its survival and transmission potential throughout the year. Thus, *H. flava* may act as an active SFTSV reservoir in winter, potentially transmitting it to other vectors like *H. longicornis* in spring and retrieving it in autumn (30).

In East Asia, *H. longicornis* is a dominant hard tick species that harbors SFTSV (10, 11) and is considered the principal intermediate vector in SFTSV transmission. The global spread of species like *H. longicornis* raises public health concerns due to their proven capacity to transmit viral and bacterial pathogens, emphasizing the need for continuous, extensive surveillance (31, 32). Although our findings align with those of previous studies reporting low SFTSV infectivity in ticks (33, 34), year-round tick surveillance revealed distinct temporal distributions for *H. flava* and *H. longicornis*, which are closely related phylogenetically (35). Notably, our study demonstrated the prevalence of *H. flava* in colder months (October to February) and the dominance of *H. longicornis* in warmer months (April to September), with similar MIR proportions (0.63% and 0.6%, respectively) (Table 4). The significant ability of *H. flava* to effectively amplify and spread the virus has been demonstrated (36). Thus, although *H. longicornis* is recognized as a major summer vector, increasing the risk to humans (37, 38), the role of *H. flava* as a virus reservoir in winter has been underestimated. The coexistence of these two tick species in the wild, facilitating SFTSV transmission, suggests the circulation of the virus throughout the year, posing an elevated threat to public health.

The prevalence of tick-borne diseases is profoundly influenced by interactions among hosts, pathogens, tick populations, and human behaviors (39). Human encroachment into wilderness areas and outdoor activities alters landscapes and faunal associations, increasing exposure risks to arthropod vectors and fostering conditions conducive to vector-borne infections. Thus, tick-borne diseases emerge at the One Health interface, intertwining human, wildlife, and ecosystem health. Additionally, climate change, altering environmental conditions conducive to tick survival, may increase tick populations and expand their geographical range (40). The effects of climate change such as longer and warmer seasons provide a more suitable environment for these tick vectors, which correlates to the increasing tick populations, and a wider range of geographical expansion. However, ticks often receive less attention than mosquitoes in vector control programs (41), potentially leading to increased tick-borne disease incidence. Ongoing surveillance is essential to map the distribution of *Haemaphysalis* ticks and other species, identify associated pathogens, and assess their public health impact. An aspect we were limited by in our study was that ticks used in this study were collected in South Chungcheong Province, and the data in our study are reflective of the phenomenon happening in this region. Although a relatively small country, South Korea exhibits diverse climatic conditions across its region, which may influence the seasonal dynamics, prevalence, and distribution of tick species. Future work is needed regarding tick sampling of wild animals from other regions of South Korea that would substantiate the seasonal dynamics of Haemaphysalis ticks. Furthermore, the collection of ticks from wild boar was not conducted during the earlier months of 2023 due to hunting restrictions. Although our findings on the relative prevalence of tick species infesting water deer and wild boar suggest that this did not significantly affect the overall outcome (Table S4), further studies are warranted to determine tick abundance in this mammalian host. This vital information enriches our understanding of SFTSV eco-epidemiology by providing spatiotemporal distribution estimates and prevalence among field-collected ticks, thereby offering independent data for assessing human encounters with infected tick.

## Data Availability

The data that support the findings of this study are available from the corresponding author upon reasonable request.

## SUPPLEMENTAL MATERIAL

Supplemental material spectrum.00489-24-s0001.pdf Tables S1 to S4.

ASM does not own the copyrights to Supplemental Material that may be linked to, or accessed through, an article. The authors have granted ASM a non-exclusive, world-wide license to publish the Supplemental Material files. Please contact the corresponding author directly for reuse.
