# Supplementary material for: Seasonal dynamics of Haemaphysalis tick species as SFTSV vectors in South Korea
Source: Microbiol Spectr. 2024 Sep 30;12(11):e00489-24. doi: 10.1128/spectrum.00489-24 (PMC11537100; doi:10.1128/spectrum.00489-24)
Supplement: Supplemental material — Tables S1 to S4. [file spectrum.00489-24-s0001.pdf]

Table S1. BLAST similarity analysis of tick species-specific conserved region (ITS1) PCR amplicons

Target gene	Closest related sequence	Contig length (nt)	BLAST percent identity
***H.longicornis,* ITS1**	*H.longicornis isolate Henan-2; ITS1* (JQ737114.1)	1021	98-100%
***H.flava,* ITS1**	*H.flava isolate 9; ITS1* (KY290814.1)	521	98-100%

Table S2. Wild animal Captured in South Korea from January to December 2023

Species	Number of Captured water deer and wild boars												
	Jan.	Feb.	Mar.	Apr.	May.	Jun.	Jul.	Aug.	Sep.	Oct.	Nov.	Dec.	Total
Water deer *(Hydropotes inermis*)	14	19	18	17	28	15	25	14	11	10	16	10	197
Wild boar *(Sus scrofa)*	0	0	0	0	0	0	0	5	0	10	10	10	35
**Total**	14	19	18	17	28	15	25	19	11	20	26	20	232

Table S3. Total ticks per host collected in South Korea from January to December 2023

Species	Stage	Number of Collected Ticks per host												
		**Jan.**	**Feb.**	**Mar.**	**Apr.**	**May.**	**Jun.**	**Jul.**	**Aug.**	**Sep.**	**Oct.**	**Nov.**	**Dec.**	**Total**
H.longicornis	Adult	0	0	2.7	6.9	8.8	82.1	60.4	24.6	10	1.7	0.9	0	198.1
	Nymph	0	2.1	16.1	38.8	13.8	4.5	3.8	11.6	75.2	18.0	1.3	0	185.2
H.flava	Adult	35.2	28.0	17.4	3.1	0.4	0	0	0.1	7.8	24.2	18.3	21.5	156
	Nymph	0	0	0	0	0	0	0	0	5.1	8.7	13.4	1.3	28.5
I.nipponensis	Adult	0	0.5	0	0	0	0	0	0	0	0.5	1.1	0.6	2.7
	Nymph	0	0	0	0.2	0	0	0	0	0	0	0	0	0.2
**Total**		35.2	30.6	36.2	49	23	86.6	64.2	36.3	98.1	53.1	35	23.4	570.7

Table S4. Ticks collected from August to December 2023 and the composing ratio of each tick species per host

Species	August		October		November		December		TOTAL	
	Wild Deer	Wild Boar	Wild Deer	Wild Boar	Wild Deer	Wild Boar	Wild Deer	Wild Boar	Wild Deer	Wild Boar
H. longicornis	598 (42.7,100%)[a]	93 (18.6, 100%)	55 (5.5, 13.1%)	341 (34.1, 52.7%)	33 (2.0, 12.5%)	19 (1.9, 5.9%)	0	0	686 (50.2, 40.8%)	453 (54.6, 38.9%)
H. flava	0	0	357 (35.7, 85.4%)	302 (30.2, 46.6%)	215 (13.4, 81.4%)	287 (28.78, 89.4%)	220 (22.0, 100%)	236 (23.6, 95.1%)	792 (71.1, 57.8%)	825 (82.5, 58.8%)
I. nipponensis	0	0	6 (0.6, 1.4%)	4 (0.4, 0.6%)	16 (1, 6%)	15 (1.5, 4.6%)	0	12 (1.2, 4.8%)	22 (1.6, 1.3%)	31 (3.1, 2.2%)

[a] Numbers in parentheses indicate the number of ticks per host and the composing ratio.
